# Biosynthesis of Iron Oxide Nanoparticles: Physico-Chemical Characterization and Their In Vitro Cytotoxicity on Healthy and Tumorigenic Cell Lines

**DOI:** 10.3390/nano12122012

**Published:** 2022-06-10

**Authors:** Elena-Alina Moacă, Claudia Geanina Watz, Daniela Flondor (Ionescu), Cornelia Păcurariu, Lucian Barbu Tudoran, Robert Ianoș, Vlad Socoliuc, George-Andrei Drăghici, Andrada Iftode, Sergio Liga, Dan Dragoș, Cristina Adriana Dehelean

**Affiliations:** 1 Department of Toxicology and Drug Industry, Faculty of Pharmacy, “Victor Babes” University of Medicine and Pharmacy Timisoara, 2nd Eftimie Murgu Square, RO-300041 Timisoara, Romania; alina.moaca@umft.ro (E.-A.M.); draghici.george-andrei@umft.ro (G.-A.D.); andradaiftode@umft.ro (A.I.); sergio.liga96@gmail.com (S.L.); dan.dragos@umft.ro (D.D.); cadehelean@umft.ro (C.A.D.); 2 Research Centre for Pharmaco-Toxicological Evaluation, “Victor Babes” University of Medicine and Pharmacy, 2nd Eftimie Murgu Square, RO-300041 Timisoara, Romania; 3 Department of Pharmaceutical Physics, Faculty of Pharmacy, “Victor Babes” University of Medicine and Pharmacy Timisoara, 2nd Eftimie Murgu Square, RO-300041 Timisoara, Romania; 4 Faculty of Industrial Chemistry and Environmental Engineering, Politehnica University Timisoara, Victoriei Square no. 2, RO-300006 Timisoara, Romania; cornelia.pacurariu@upt.ro (C.P.); robert_ianos@yahoo.com (R.I.); 5 Electron Microscopy Laboratory “Prof. C. Craciun”, Faculty of Biology & Geology, “Babes-Bolyai” University, 5-7 Clinicilor Street, RO-400006 Cluj-Napoca, Romania; lucianbarbu@yahoo.com; 6 Electron Microscopy Integrated Laboratory, National Institute for R & D of Isotopic and Molecular Technologies, 67-103 Donat Street, RO-400293 Cluj-Napoca, Romania; 7 Romanian Academy—Timisoara Branch, Center for Fundamental and Advanced Technical Research, Laboratory of Magnetic Fluids, 24 M. Viteazu Ave., RO-300223 Timisoara, Romania; vsocoliuc@gmail.com

**Keywords:** green synthesis, *Artemisia absinthium* L., iron oxide nanoparticles, in vitro screening

## Abstract

Iron oxide nanoparticles were synthesized starting from two aqueous extracts based on *Artemisia absinthium* L. leaf and stems, employing a simplest, eco-friendliness and low toxicity method—green synthesis. The nanoparticles were characterized by powder X-ray diffraction (XRD), Fourier transformed infrared spectroscopy (FT-IR), X-ray fluorescence analysis (XRF), thermal analysis (TG/DSC), and scanning electron microscopy (SEM). Lack of magnetic properties and the reddish-brown color of all the samples confirms the presence of hematite as majority phase. The FTIR bands located at 435 cm^−1^ and 590 cm^−1^, are assigned to Fe-O stretching vibration from hematite, confirming the formation of α-Fe_2_O_3_ nanoparticles (NPs). The in vitro screening of the samples revealed that the healthy cell line (HaCaT) presents a good viability (above 80%) after exposure to iron oxide NPs and lack of apoptotic features, while the tumorigenic cell lines manifested a higher sensitivity, especially the melanoma cells (A375) when exposed to concentration of 500 µg/mL iron oxide NPs for 72 h. Moreover, A375 cells elicited significant apoptotic markers under these parameters (concentration of 500 µg/mL iron oxide NPs for a contact time of 72 h).

## 1. Introduction

Green nanotechnology is based on the use of green nanomaterials, safe for human health or nanoproducts designed to provide solutions for medical system as well as for the environmental problems [1,2,3,4,5]. Of late years, there has been a growing interest regarding nanomaterials due to their commercial applications in many industries [6,7,8,9]. Iron oxide-based nanomaterials have become more common and used, due to their unique properties, among which the most important feature is the biocompatibility with human body and the environment, as well as excellent stability under ambient conditions [10]. Iron oxides nanoparticles (IONPs) are of great interest, being available in nature as well as easily synthesized in laboratory. The different polymorph forms of iron oxide (magnetite (Fe_3_O_4_), maghemite (γ-Fe_2_O_3_), and hematite (α-Fe_2_O_3_) are of technological and medical importance [11,12].

Hematite (α-Fe_2_O_3_) is the most chemically and thermodynamically stable at room temperature of all iron oxides, with a corundum crystal structure, weakly ferromagnetic or antiferromagnetic properties, but very widespread in rocks and soils [13]. Nevertheless, hematite has been widely used for his electric activity (photo-catalyst, pigment, gas sensor, oxidizer in thermite composition, semiconductor), due to its low cost and high resistance to corrosion [14,15,16,17]. In addition, hematite can be used as a precursor for the synthesis of γ-Fe_2_O_3_ and Fe_3_O_4_ [18] and in eradication of environment pollution (as adsorbent for poisons from contaminated water) [19,20]. Regarding the biomedical potential of hematite, the scientific literature has already reported several applications of α-Fe_2_O_3_ as a treatment option in several specific tumors and cancers [21,22,23,24,25,26].

Chemical and physical routes are available to produce iron oxides nanoparticles, yet, these methods involve the use of toxic, corrosive or flammable raw materials, which after chemical reaction, leave behind hazardous by-products that affect both the environment and human health. The conventional methods used for the synthesis of α-Fe_2_O_3_ are as follows: chemical co-precipitation, thermal decomposition, solvothermal and hydrothermal methods, sonochemical method, sol-gel reactions, microemulsion method, laser pyrolysis, combustion method [27,28,29] etc.

However, employing these types of methods requires high cost equipment, high temperature and pressure, high energy, and/or vacuum. Thereby, nanoscience is increasingly focusing on the development of environment-friendly routes for the synthesis of IONPs. In this regard, various biological entities (bacteria, fungi, algae, plant extracts, actinomycetes, plant biomasses) are considered potential alternatives for obtaining IONPs. Plant-mediated green synthesis is a cost-effective, simple, inexpensive, biocompatible, and ecofriendly method, which is based on the use of plant extract rich in phytochemical compounds of medicinal importance, which acts both as reducing and stabilizing agent [30]. All the constituents present in the plant extract (antioxidants, flavonoids, terpenoids, phenolic compounds etc.,) play an important role in biosynthesis of IONPs. Many studies have reported the synthesis of α-Fe_2_O_3_ nanoparticles using plant extracts, for example: *Melaleucane sophila* [31], *Eucalyptus leaf* [32], *Tridax procumbens* [33], Callistemon viminalis [34] *Rosmarinus officinalis* [35], *Sida cordifolia* [36], *Arisaema amurense* root [22], *Guava (Psidium guajava)* leaves [37], *Henna (Lawsonia inermis)* leaf [38], *Pistachio* leaf (*Pistacia vera* L.) [39], *Salvadora persica* [25], *Green Tea (Camellia sinensis)* leaves [40], *Ailanthus excelsa* leaves [41], *Azadirachta indica* leaf [42], *Moringa oleifera* leaf [43], *Laurus nobilis* L. [44], *Cornus mas* L. [45], *Platanus orientalis* leaf [46], *Rhus punjabensis* [23], and many more. Basavegowda and coworkers reported a successful study regarding the use of *Artemisia annua* aqueous extract based on leaves, for the synthesis of magnetite (Fe_3_O_4_) nanoparticles. The authors used the pre-formed Fe_3_O_4_ nanoparticles as a catalyst for the preparation of benzoxazinone and benzthioxazinone derivatives, with multiple pharmacological and biological activities [47].

To the best of our knowledge, no studies have been reported on the synthesis of Fe_2_O_3_ nanoparticles starting from aqueous extract of any species of *Artemisia* based on leaves and stems. Therefore, the purposes of the present study are: (i) the development of iron oxide nanoparticles through the green method starting from two aqueous extracts of *Artemisia absinthium* L., obtained from leaves and stems; (ii) the physico-chemical screening of the pre-formed iron oxide nanoparticles; (iii) the in vitro biological assessment of both extracts of *Artemisia absinthium* L. and of the iron oxide nanoparticles resulting from them. In this regard, the in vitro model employed was based on three different cell lines: HaCaT–immortalized human keratinocytes, A375–human amelanotic melanoma, and A431–human carcinoma cell line.

## 2. Materials and Methods

### 2.1. Preparation of Leaf and Stems Aqueous Extracts

*Artemisia absinthium* L. was collected from local fields of Vâlcea County (Southeast of Romania) and was taxonomically identified by Prof. Dr. Diana Antal from the Department of Pharmaceutical Botany of our University. The whole plant was washed and dried in an oven, at 23 ± 1 °C (ED 260, Binder GmbH, Tuttlingen, Germany) for 3 days and then each component part of the plant was collected separately (leaf and stems) and crushed until a fine powder was obtain. Both aqueous extracts were obtained using the maceration method, by mixing 25 g of powder’s leaves and stems with 500 mL distilled water, stirred steadily for 24 h at room temperature and then boiled for 1 h. After cooling, both aqueous extracts were filtered through a Whatman no. 42 filter paper and concentrated under reduced pressure at 40 °C using a rotary evaporator (Heidolph G3, Schwabach, Germany). Each crude extract was subsequently lyophilized and stored at 4 °C in a glass container until further use.

### 2.2. Green Synthesis of Iron Oxide Nanoparticles

**Figure 1:**
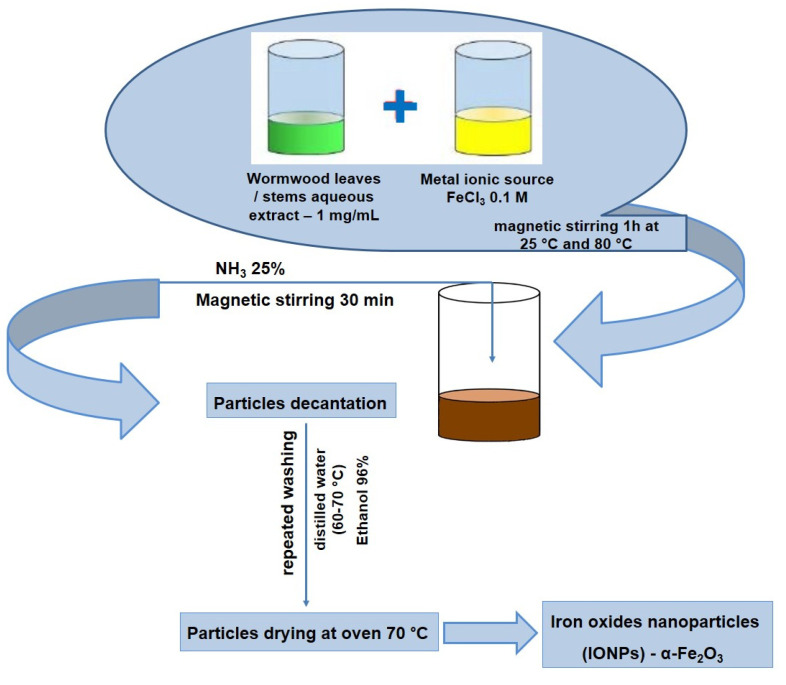
Schematic protocol of iron oxide nanoparticles synthesis by green method.

Aliquots of both lyophilized extracts were dispersed in ultrapure water (Milli-Q^®^ Integral Water Purification System (Merck Millipore, Darmstadt, Germany)), by sonication process (50% amplitude for 10 min; UP200S from Hielscher Ultrasonics GmbH, Teltow, Germany). Iron oxide nanoparticles were prepared by a modified protocol from previous studies [47,48,49], by adding 0.1 M FeCl_3_ (Merck, Darmstadt, Germany) solution to each wormwood extract (W) in a 1:1 volume ratio. After magnetic stirring at different temperatures, the mixtures were precipitated with 25% NH_4_OH (Chemical Company SA, Iasi, Romania) and the iron oxides nanoparticles (IONPs) were immediately obtained. The formed IONPs were further left to magnetic stirring for another 30 min, washed with distilled water and ethanol 96% (Chemical Company SA, Iasi, Romania) for several times in order to remove the impurities and unreacted salt and dried at 70 °C in an oven (POL-EKO Aparatura, Wodzisław Slaski, Poland). Four types of nanoparticles were prepared using different synthesis condition: from wormwood leaves (WL)—when the precipitation was made at 25° (WL2) and at 80 °C (WL1); and from wormwood stems (WS) respectively—when the precipitation was made at 25° (WS2) and at 80 °C (WS1). A schematic protocol is depicted in Figure 1.

### 2.3. Physico-Chemical Characterization

After preparation, all the four IONPs were submitted to structural characterization by X-ray diffraction (XRD), in order to evaluate the phase composition. The XRD analysis was performed using the Rigaku Ultima IV instrument (Tokyo, Japan). The operating parameters set were 40 kV and 40 mA and the XRD pattern was recorded using CuKα radiation (λ = 0.15406 nm) with 2θ scan at room temperature, at a lower scan rate (2 degrees/minute). The following PDF files were used for peak assignment: 330664 (α-Fe_2_O_3_); 391346 (γ-Fe_2_O_3_) and 190629 (Fe_3_O_4_) from the International Centre for Diffraction Data Powder Diffraction File (ICDD PDF) 4+ 2019 data.

In order to assess the stability of organic compounds from wormwood aqueous extract which probably exist on the IONPs surface, the samples were analyzed in terms of heating behavior, within the range of 10 to 1000 °C by thermal analysis using a Netzsch STA 449 C instrument (Selb, Germany). Aluminum crucibles were used to record the thermogravimetric (TG) and differential scanning calorimetry (DSC) curves, under artificial air flow of 20 mL/min at a heating rate of 10 °C/min.

Regarding the identification of the functional groups on the IONPs surface, the FTIR spectra were recorded, using a Shimadzu Prestige-21 spectrometer (Duisburg, Germany) within the range of 400 to 4000 cm^−1^, using KBr pellets and 4 cm^−1^ resolution.

By scanning electron microscopy (SEM) was assess the morphology and ultrastructure of IONPs. SEM analysis was carried out using a Hitachi SU8230 cold field emission gun STEM (Chiyoda, Tokyo, Japan) microscope with EDX detectors X-Max^N^ 80 from Oxford Instruments (UK). The operating parameters set were high-vacuum (HV mode), acceleration voltage-30 kV, secondary electron detectors (upper and lower), with two magnification orders, one for a general overview of the image/measurements and another for higher surface topography for regions of interest. The identified chemical species were expressed in atomic percent (At %).

Transmission electron microscopy (TEM) was employed to evaluate the particle size, using a Hitachi HD2700 cold field emission gun STEM (Chiyoda, Tokyo, Japan) equipped with two windowless EDX detectors (X-MaxN 100). By using ImageJ (https://imagej.nih.gov/ij/, accessed on 24 May 2022), the IONPs size and aspect ratio statistics were determined from SEM and TEM images.

By using a Zatasizer Nano ZS from Malvern Instruments (Worcestershire, UK), the hydrodynamic diameter (Hd) and polydispersity index (PDI) were assessed. The colloidal particles size were measured in aqueous suspension, through photon correlation spectroscopy, in a range between 0.4 nm and 9 µm, at 37 °C.

Traces of metal were determined by X-ray fluorescence (XRF) analysis, using an X-MET8000 series, HHXRF models spectrometer (Hitachi, Chiyoda, Japan), with a wavelength dispersive X-ray fluorescence. The IONPs obtained by green synthesis were measured with the portable stand for complete protection of the user against scattered radiation, according to the literature [50,51,52]. The elementary composition of the measured sample can be calculated based on the principles of physics.

### 2.4. In Vitro Model–Cell Culturing Protocol

The in vitro model employed in the current study consisted in one healthy cell line: HaCaT–immortalized human keratinocytes (code no. 300493; CLS Cell Lines Service GmbH) and two tumorigenic cell lines purchased from American Type Culture Collection (ATCC): A375–amelanotic human malignant melanoma (code no. CRL-1619) and A431–epidermoid human carcinoma (code no. CRL-1555). The culture medium used for cell growth was Dulbecco’s modified Eagle’s medium with high glucose (concentration of 4.5 g/L) and 15 mM HEPES (ATCC, code no. DMEM 30-2002™), enriched with 10% fetal calf serum and 1% antibiotic mixture of 100 U/mL penicillin and 100 µg/mL streptomycin. The antibiotic mixture was added as a safety measure to avoid any possible microbiologic infection. All in vitro procedures were performed under sterile conditions, using a biosafety cabinet (MSC Advantage 12 model from ThermoFisher Scientific, Inc., Waltham, MA, USA).

### 2.5. Alamar Blue Assay–Cell Viability Assessment

The Alamar Blue colorimetric test was performed to quantify the cell viability percentage of immortalized human keratinocytes-HaCaT, human amelanotic malignant melanoma cell line-A375, and epidermoid human carcinoma cells-A431, after exposure to two different types of *Artemisia absinthium* L. extracts (a—leaves extract (WL) and b—stems extract (WS) and different iron oxide NPs obtained from these extracts (WL 1_Fe_2_O_3_ NPs; WL 2_Fe_2_O_3_ NPs, respectively WS 1_Fe_2_O_3_ NPs; WS 2_Fe_2_O_3_ NPs). The incubation period used for this technique was 24 h, 48 h, and 72 h. The Alamar Blue colorimetric test was employed, as previously described [53]—in brief, 1 × 10^4^ cells/well were seeded onto 96-well culture plates and incubated at 37 °C and 5% CO_2_, until a confluence of approximately 80% was reached. After this step, the old medium was replaced with cell culture medium containing three different concentrations (150, 300, and 500 µg/mL) of test samples. The test concentrations (150, 300, and 500 µg/mL) were obtained from a stock solution of 5 mg IONPs/mL miliQ water, that was ultrasonicated at 50% amplitude, for 10 min (total energy–30,152 J), using a QSonica Ultrasonic Liquid Processor 700 W, Q700 Sonicator (Newtown, CT, USA)). The control cells were treated with cell culture medium and maintained under identical conditions as the test-treated cells. To quantify the viable cell population, the absorbance of the wells (control and sample-treated cells) was determined spectrophotometrically by reading the absorbance at two different wavelengths (570 nm and 600 nm) using a microplate reader (xMarkTM Microplate, Bio-Rad Laboratories, Hercules, CA, USA).

### 2.6. LDH Release Method–Cytotoxicity Test

The cytotoxic rate of the test samples was determined after a period of 72 h by quantifying the LDH leakage into the extracellular media. The protocol employed for LDH quantification was similar to the one used for the Alamar Blue test. However, on the day of the assay, 50 µL medium/well was transferred to a new 96-well plate and mixed with 50 µL reaction mixture, followed by an incubation of 30 min at room temperature. Afterwards, 50 µL stock solution/well was added and the absorbance of each well was determined spectrophotometrically at the wavelengths of 490 nm and 680 nm by means of a microplate reader (xMarkTM Microplate, Bio-Rad Laboratories, Hercules, CA, USA).

### 2.7. 4′,6- Diamidino-2-Phenylindole (DAPI) Staining–Evaluation of Apoptosis Markers

HaCaT and A375 cell lines were cultured on coverslips using an initial density of 3 × 10^5^ cells/well, using 6-well cell culture plates. After 24 h, the cells were treated with test compounds (WL extract; WS extract and WL 1_Fe_2_O_3_ NPs; WL 2_Fe_2_O_3_ NPs, respectively WS 1_Fe_2_O_3_ NPs; WS 2_Fe_2_O_3_ NPs) at concentrations of 500 μg/mL for an interval of time of 72 h. At 72 h post-treatment, the staining protocol was performed by employing the following steps: fixation of the cell culture by using 4% paraformaldehyde in PBS; permeabilization with 2% Triton-X in PBS; followed by a blocking steps that consists in addition of 30% FCS in 0.01% Triton-X. In the end, the culture cells were washed with PBS and stained by using a concentration of 300 nM 4′,6-diamidino-2-phenylindole (DAPI) for 15 min. All images were taken using the integrated DP74 digital camera of an Olympus IX73 inverted microscope (Olympus, Tokyo, Japan).

### 2.8. Statistical Analysis

GraphPad Prism 9.3.0 version (GraphPad Software, San Diego, CA, USA) was used for data collection and statistical analysis. Data are presented as mean of three independent experiments ± standard deviation (SD). One-way ANOVA was employed to determine the statistical differences, followed by Dunnett’s multiple comparisons post-test.

## 3. Results and Discussion

Every day nanoscience advances in the production of nanoparticles, environmentally friendly, with a dimension less than 100 nm, with multiple biological and industrial properties. In the last decades, many researchers were focused on design strategies of multifunctional IONPs with application on biomedical domain, by combining them with bioactive constituents aiming to enhance their biomedical potential. However, the most handy, cheap and natural bioactive constituents are found in plants, thereby the simplest method by which IONPs with bioactive constituents can be obtained is the green synthesis, where the active phytochemicals (antioxidants, flavonoids, terpenoids, tannins, vitamins or phenolic compounds) from plants act as reducing (for iron salts precursor) and stabilizing agents in the formation of nanoparticles. Moreover, they adhere to the surface of IONPs, thus being responsible for the biological efficacies [30,54,55].

In this regard, the purpose of the present study was, in addition to obtaining different iron oxide NPs through green synthesis pathway using an aqueous plant extract (leaves and stems wormwood extracts of *Artemisia absinthium* L.) as reducing agent, a complex physicochemical screening of the sample by employing XRD, TG-DSC, FTIR, SEM-EDX, TEM, and XRF analysis in order to confirm the chemical composition and the physicochemical features of the newly synthetized iron oxide NPs.

### 3.1. Physico-Chemical Characterization of IONPs

#### 3.1.1. Structural IONPs Characterization (XRD)

**Figure 2:**
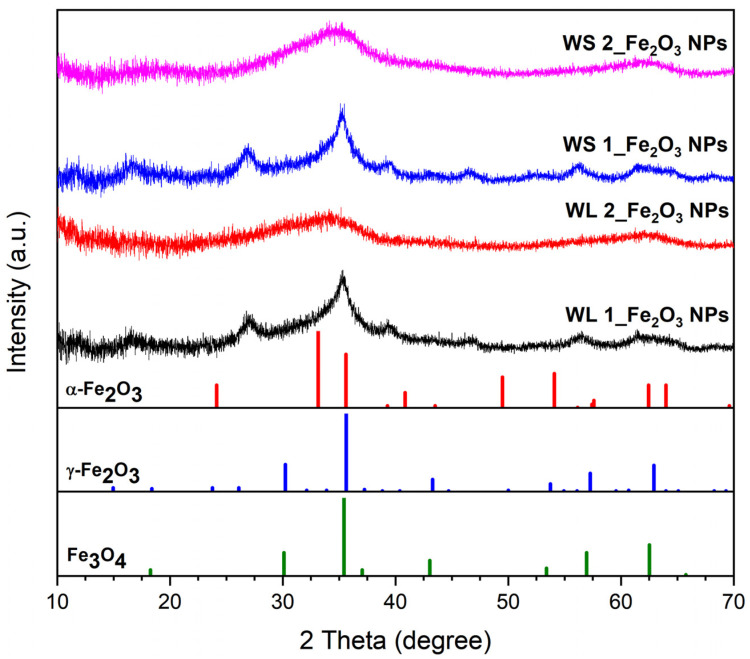
XRD patterns of IONPs obtained from wormwood leaves and stems aqueous extracts versus XRD patterns of hematite–α-Fe_2_O_3_ (red, PDF file: 330664), maghemite–γ-Fe_2_O_3_ (blue, PDF file: 391346), and magnetite–Fe_3_O_4_ (green, PDF file: 190629) from (ICDD PDF) 4+ 2019 data.

Figure 2 exhibits the XRD patterns of IONPs synthesized by green method, starting from wormwood leaves aqueous extract (WL 1–with 0.1 M FeCl_3_ salt precipitation at 80 °C and WL 2–with 0.1 M FeCl_3_ salt precipitation at 25 °C) and from wormwood stems aqueous extract (WS 1–at 80 °C and WS 2–at 25 °C).

The XRD patterns of the samples prepared at 25 °C (WL 2_Fe_2_O_3_ NPs and WS 2_Fe_2_O_3_ NPs) suggest the samples are practically amorphous, hence the lack of diffraction peaks, due to the fact that the amorphous samples show no diffraction peak. Samples prepared at 80 °C (WL 1_Fe_2_O_3_ NPs and WS 1_Fe_2_O_3_ NPs) show a very small-intensity and broad diffraction peak at around 2 Theta = 35°, which indicates the presence of very small crystallites, which is similar to other patterns reported in the literature [56,57] and assigned to maghemite (γ-Fe_2_O_3_) nanoparticles. Taking into account the fact that the maximum diffraction positions of Fe_3_O_4_ and γ-Fe_2_O_3_ nanoparticles are very close, according to the PDF files 190629 and 391346, therefore, in the case of samples WL 1_Fe_2_O_3_ NPs and WS 1_Fe_2_O_3_ NPs the presence of both compounds cannot be excluded. It is most likely that each sample contains a mixture of hematite (predominant phase due to lack of magnetic moment) and/or maghemite/magnetite (probably in traces). The aspect of all samples are reddish-brown and extremely weak magnetic when operated with an external block neodymium magnet (NdFeB; Q-60-30-15-N, www.supermagnete.ro, accessed on 2 December 2021). Due to the fact that all the samples are practically amorphous, one cannot say exactly whether, in addition to hematite, there is magnetite or maghemite. In addition, given the actual profile of the XRD patterns one cannot fully assess the samples composition, but, considering the additional information available (the brownish color of the samples, extremely weak sample attraction by the magnetic field) one may assume the presence of magnetite (Fe_3_O_4_) and maghemite (γ-Fe_2_O_3_) alongside hematite (α-Fe_2_O_3_). Our results regarding the XRD profile are in accordance with the literature data [58,59,60].

#### 3.1.2. Thermal Behavior

**Figure 3:**
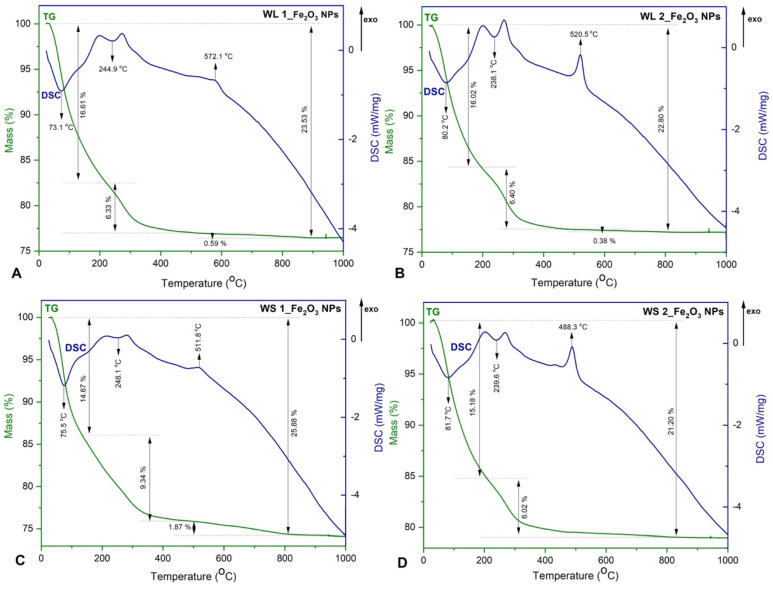
TG−DSC curves IONPs obtained by green synthesis; (**A**) Fe_2_O_3_ NPs from WL aqueous extract at 80 °C, (**B**) Fe_2_O_3_ NPs from WL aqueous extract at 25 °C, (**C**) Fe_2_O_3_ NPs from WS aqueous extract at 80 °C, (**D**) Fe_2_O_3_ NPs from WS aqueous extract at 25 °C.

In Figure 3 is depicted the TG-DSC graphic of IONPs obtained by green synthesis.

The TG−DSC curves indicate there are no significant differences between IONPs samples. The endothermic/exothermic effects recorded on all DSC curves are more intense when the precipitation was made at room temperature (25 °C) (Figure 3B,D). According to the TG analysis (Figure 3), the total weight loss percentage in all samples was about 23 ± 2%. The highest weight loss was recorded below 400 °C (~20%), attributed to the water molecules loss of the phytocompounds on the IONPs surface. In addition, between 200 and 300 °C the DSC curves show two overlapped exothermic effects, probably related to the oxidation of organic compounds from the plant extract.

After 400 °C, on TG graphics (Figure 3A–C), the weight loss was about 1 ± 0.5%. This weight loss could be considered negligible and the phenomenon occurred is assigned to phase transformation of secondary phases obtained (γ-Fe_2_O_3_ and/or Fe_3_O_4_) into α-Fe_2_O_3_ [23,37]. At 488.3 °C a phase transformation occurs, without mass loss (Figure 3D). This phase transformation is characteristically to the transformation of γ-Fe_2_O_3_ to α-Fe_2_O_3_. In addition, the reddish-brown color of IONPs could be an excellent indicator in formation of α-Fe_2_O_3_ [61].

Moreover, due to the fact that on the temperature range 400–1000 °C occurs a much lower weight loss, this fact assures the complete formation of α-Fe_2_O_3_. Regarding the DSC curves, exothermic and endothermic peaks were recorded. Forasmuch as neither exothermic effect accompanied with mass growth on TG curve, was detected, confirm that in all sample it was obtained only hematite as a majority phase and traces of maghemite. If the exothermic effect could have been detected around 200–250 °C with a growth mass, we could have said that in the samples we also had magnetite, which at this temperature oxidizes at maghemite. The exothermic effects recorded around 500 °C, without mass loss (or negligible), confirms the maghemite transition to hematite [62,63].

#### 3.1.3. FTIR Investigations

**Figure 4:**
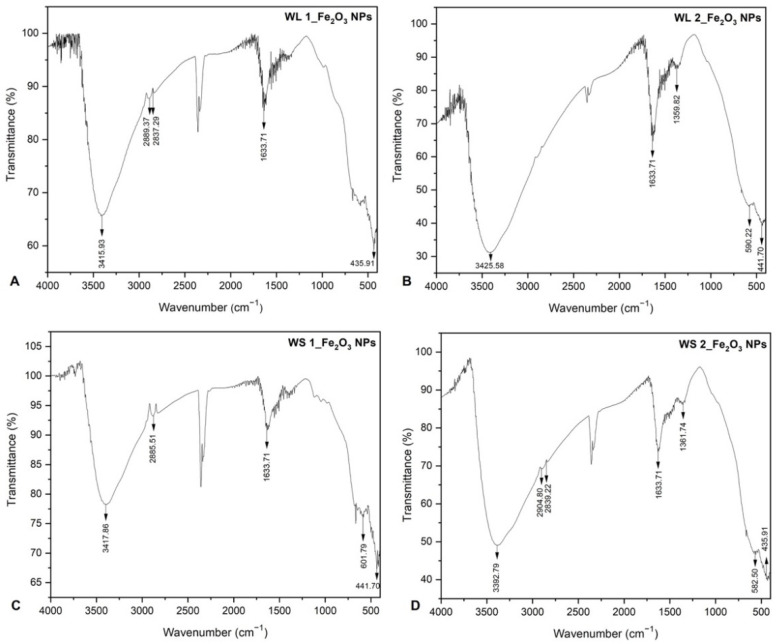
FTIR spectrum of IONPs obtained by green synthesis; (**A**) Fe_2_O_3_ NPs from WL at 80 °C, (**B**) Fe_2_O_3_ NPs from WL at 25 °C, (**C**) Fe_2_O_3_ NPs from WS at 80 °C, (**D**) Fe_2_O_3_ NPs from WS at 25 °C.

Figure 4 shows the FTIR spectra of all the IONPs samples, obtained by green synthesis. FTIR experiments were carried out in order to identify the presence of phytocompounds on the surface of iron oxide nanoparticles, come from the leaves and stems wormwood, as well as their role in reduction of iron ions from FeCl_3_. As can be noticed, there are no significant differences between the absorption bands of each sample. The band located around 3400 cm^−1^ can be attributed to the O-H stretching vibration of hydroxyl group. The adsorption peaks which denote the presence of hydroxyl groups (O-H), are assumed to be the phytocompounds responsible with the reduction of iron ions from FeCl_3_ [64]. The medium band appearance present at approximately 1633 cm^−1^ could be associated to the symmetric and asymmetric bending modes of C=O bonds of amino acid and esters respectively contained in leaves and stems aqueous extracts. In addition, also these peaks located around 1633 cm^−1^, characteristics of proteins/enzymes from plant extracts, have been found to be responsible for the reduction of iron ions [65,66].

The bands located in the inorganic domain characteristic for the Fe-O vibration reveal bands that are not so well defined are located between 435.91 cm^−1^ and 601.79 cm^−1^. These bands can be assigned to the Fe-O stretching vibration from hematite with the possibility of a mixture of phases that could include maghemite or/and magnetite. The peaks recorded in the range 435–601 cm^−1^, assigned to Fe-O vibration, confirm the formation of α-Fe_2_O_3_ nanoparticles [67,68,69]. Moreover, many researchers who synthesized pure hematite starting from various plants extracts, found that the vibrational bands under 600 cm^−1^ are assigned to Fe-O nanoparticles [34,35,70].

In the case of samples WL 1_Fe_2_O_3_ NPs (Figure 4A) and WS 2_Fe_2_O_3_ NPs (Figure 4D), it is visible the formation of a doublet at around wavelength 2800–2900 cm^−1^ characteristic for the C-H stretching of alkane functional groups. Moreover, the bands which are well defined around 1350–1360 cm^−1^ (Figure 4B,D) correspond to the O-H bending of phenol functional groups, and are present in the samples due to the phenolic compounds from *Artemisia absinthium* L. aqueous extract. These phenolic groups appeared well defined only when the nanoparticles were obtained at room temperature (25 °C). These functional groups are assumed to be responsible for the capping and stabilization of the pre-formed IONPs with O-H functional groups on their surface. It is well-known that the capping agents contribute to the reduction of nanoparticles aggregation.

#### 3.1.4. SEM-EDX Analysis

**Figure 5:**
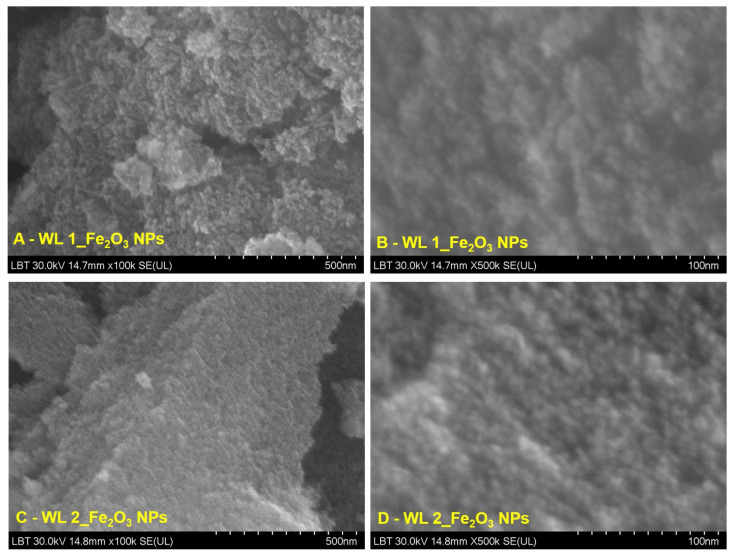
SEM images of IONPs obtained from wormwood leaves aqueous extract (WL 1–at 80 °C and WL 2–at 25 °C), at different orders of magnitude: (**A**,**C**) 500 nm scale bar, (**B**,**D**) 100 nm scale bar.

**Figure 6:**
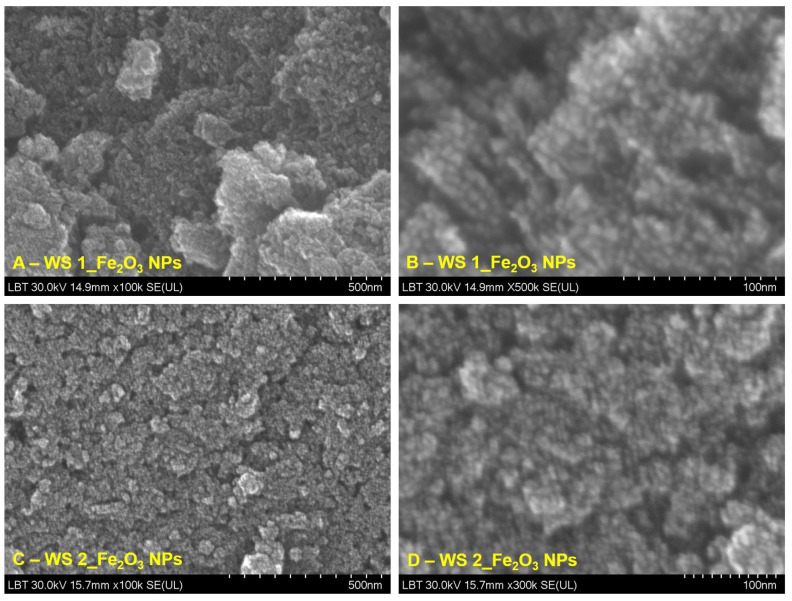
SEM images of IONPs obtained from wormwood stems aqueous extract (WS 1–at 80 °C and WS 2–at 25 °C), at different orders of magnitude: (**A**,**C**) 500 nm scale bar, (**B**,**D**) 100 nm scale bar.

Figure 5 and Figure 6 show representative images at different orders of magnitude—one image as a general overview (100 k) of each IONPs obtained starting from leaves (WL) and stems (WS) wormwood aqueous extracts and one image at higher magnification (500 k).

It can be noticed that the IONPs obtained starting from leaves wormwood aqueous extract (WL) have quasi-spherical shape, being uniformly distributed. An agglomeration of nanoparticles is observed when those were obtain at 80 °C (Figure 5B); probably due to the large surface area to volume ratio, the surface energy of nanoparticles is minimized, as compare to the sample obtained at 25 °C (Figure 5D).

Regarding IONPs obtained starting from stems wormwood aqueous extract (WS) (Figure 6), the samples appears slightly agglomerated, being more uniformly distributed, especially in the case of nanoparticles formed at 80 °C (Figure 6B). Moreover, the nanoparticles preserve the quasi-spherical shape as well as the nanometric scale.

**Figure 7:**
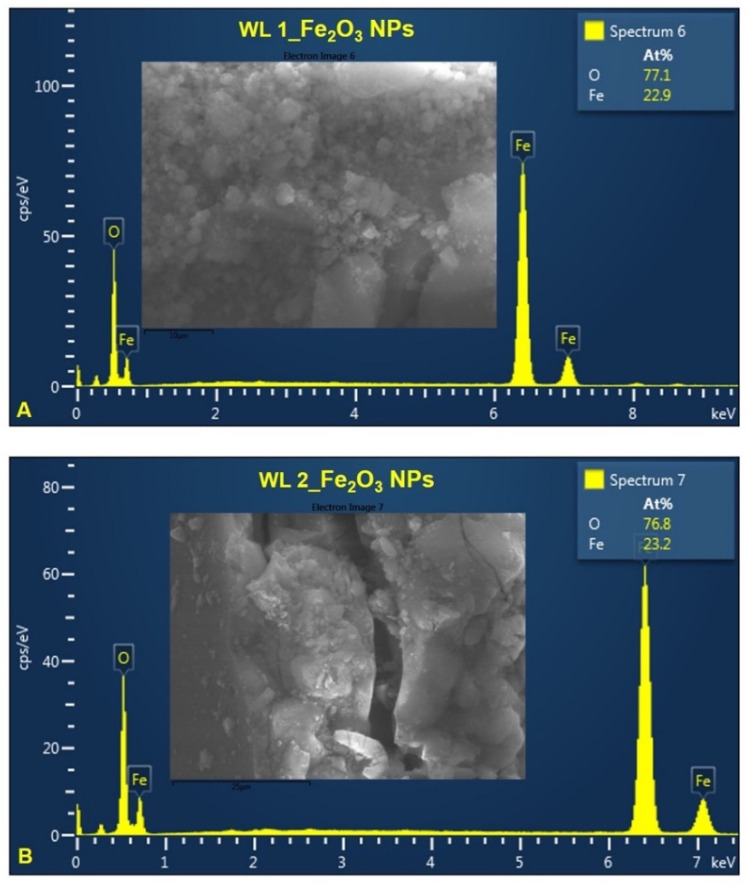
EDX spectra of IONPs obtained from WL: (**A**) Fe_2_O_3_ NPs from WL at 80 °C, (**B**) Fe_2_O_3_ NPs from WL at 25 °C.

**Figure 8:**
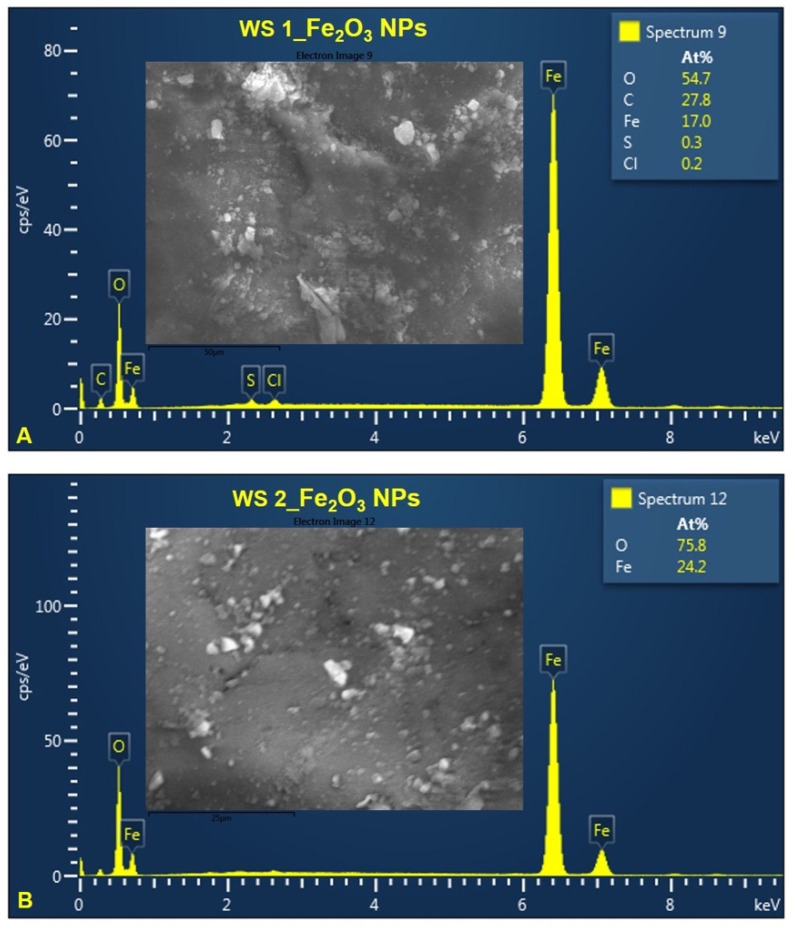
EDX spectra of IONPs obtained from WS: (**A**) Fe_2_O_3_ NPs from WS at 80 °C, (**B**) Fe_2_O_3_ NPs from WS at 25 °C.

Figure 7 and Figure 8 show the chemical composition of IONPs obtained by green synthesis. EDX analysis proves the presence of Fe and O in all synthesized samples. According to the atomic percentage values of elements, it could be observed that the amount ratio of Fe to O is approximately 2:3, which indicate that the nanoparticles are Fe_2_O_3_ (Figure 7A,B). In the case of IONPs obtained starting from stems wormwood aqueous extract, at 80 °C (Figure 8A), the amount ratio between Fe and O was not 2:3. This may be due to the high nanoparticles production temperature and the fact that the wormwood stems do not contain as many organic compounds (phenolic compounds) as the leaves, which contribute to the reduction process of the iron ions.

Regarding the study on the morphology, nanoparticles shape and elemental composition of the synthesized IONPs, the ImageJ analysis on 260 particles from SEM images, revealed that the IONPs are in nanometric domain, extremely small (4.7 ± 0.8 nm) with quasi-spherical shape of 1.14 ± 0.09 aspect ratio (Figure 5 and Figure 6). The elemental composition of the synthesized iron oxide nanoparticles, evaluated by EDX analysis (Figure 7 and Figure 8), showed that the atomic percent ratio between Fe and O is 2:3, which indicate the purity of the synthesized hematite phase of nanoparticles [71,72,73]. The carbon peak, depicted in all EDX spectra, around 1.5 keV, represent the tape used as grid support for the iron oxide nanoparticles immobilization. In the case of IONPs obtained from stems wormwood aqueous extract, at 80 °C, appear two more elements (S and Cl). Cl probably was derived from the metal salt used (FeCl_3_) and S was most likely from some impurities on the vessel in which the synthesis of IONPs was performed; but in the same time both elements could be emanated from the stems aqueous wormwood extract.

#### 3.1.5. TEM Analysis

**Figure 9:**
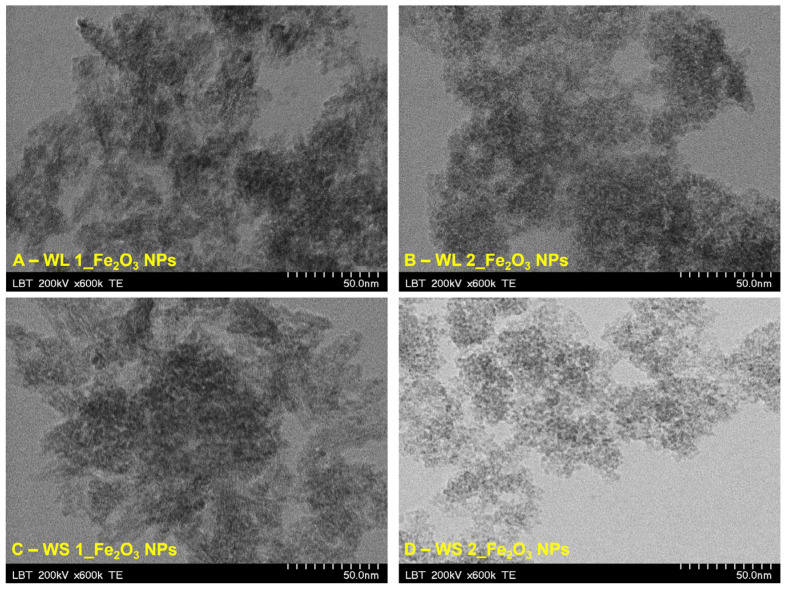
TEM images of IONPs obtained from WL aqueous extract: (**A**) Fe_2_O_3_ NPs from WL at 80 °C, (**B**) Fe_2_O_3_ NPs from WL at 25 °C and WS aqueous extract: (**C**) Fe_2_O_3_ NPs from WS at 80 °C, (**D**) Fe_2_O_3_ NPs from WS at 25 °C.

In Figure 9, are depicted the TEM images of the aqueous suspensions containing IONPs synthesized by green method starting from aqueous extract based on stems and leaves of *Artemisia absinthium* L. Regarding the aqueous suspensions with IONPs obtained from wormwood leaves (Figure 9A,B) one can observe that these are extremely agglomerated, especially the IONPs obtained at 80 °C (Figure 9A) with the particle size range between 2.9 to 3.1 nm. On the other hand, the aqueous suspensions with IONPs obtained from wormwood stems (Figure 9C,D) indicate that nanoparticles are slightly agglomerated, with the particles size range from 2.3 to 3.0 nm. Consequently, the average size of synthesized nanoparticles is consistent with the size of particles determined by XRD (the amorphous structure).

**Figure 10:**
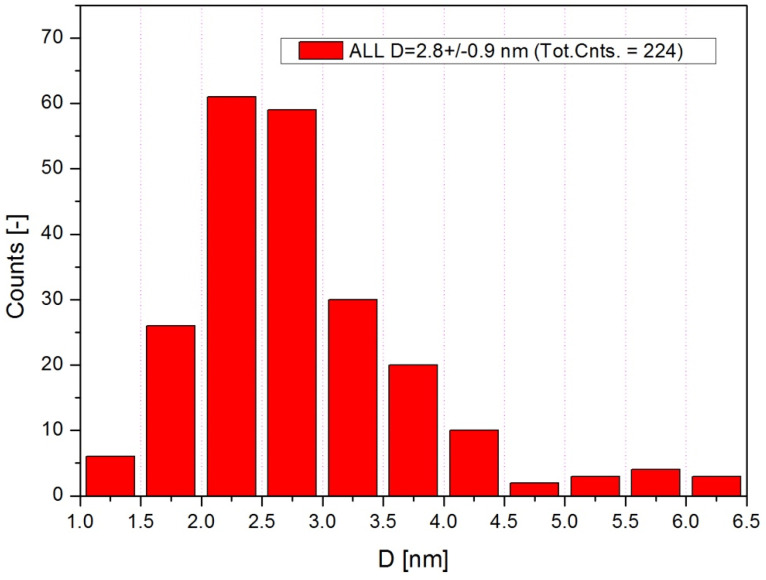
The counts distribution of particles size, from all four samples.

In Figure 10 is depicted the particle size distribution extracted from TEM images, as an average from all four samples. The particle size distribution in each sample is shown in Table 1.

One can observe a difference between the particle size determined by SEM (4.7 ± 0.8 nm) and the particle size determined by TEM (2.8 ± 0.9 nm). This difference is due to the layer of phytocompounds attached to the surface of the nanoparticles formed and it is due to the aqueous extract of wormwood leaves and stems. In addition, phytocompounds are evenly distributed on the surface of nanoparticles, because the average aspect ratio obtained from both TEM and SEM was very close: 1.14 ± 0.08 vs. 1.14 ± 0.09 respectively.

#### 3.1.6. Dynamic Light Scattering (DLS) Measurement

In Table 2 are described the results regarding the values of the hydrodynamic diameter (Hd) and polydispersity index (PDI) for all the aqueous suspensions containing IONPs from leaves and stems of wormwood extract, recorded at 37 °C.

It can be noticed that sample WL1_Fe_2_O_3_ NPs, obtained from wormwood leaves at 80 °C, revealed the highest hydrodynamic diameter ~9 µm, followed by WL 2_Fe_2_O_3_ NPs, obtained at 25 °C, with a Hd of ~1 µm, which means that the small particles are agglomerated in huge clusters. Contrarily, the aqueous suspensions containing IONPs from wormwood stems, regardless of reaction temperature, revealed an Hd of nanometric domain (244 nm for IONPs obtained at 80 °C and 309 nm for IONPs obtained at 25 °C).

The clusters of micrometer domain are observed only when the IONPs were obtained from the leaves of *Arthemisia absinthum* L. and when the temperature was increased. In this regard, one can affirm that the phytocompounds contained in wormwood leaves have a crucial role in the formation of nanoparticles with specific morphology and size. These are two important parameters that must be taken into account when preparing IONPs, alongside reaction temperature.

*Artemisia absinthum* L. (wormwood) is reported to be composed of sesquiterpenes, volatile oils, flavonoids, hydroxybenzoic acids, hydroxycinnamic acids, resveratrol and other, known for anti-inflammatory, anthelmintic, antipyretic, antibacterial, insecticide, and anticancer properties [73,74,75,76,77]. At the same time, all these phytocompounds are actively involved in the formation of iron oxide nanoparticles.

In a previous study of our team, were identified the LC-MS fingerprint as well as the FT-IR experiments from leaves and stems wormwood extracts [77]. In that research study, we found that wormwood leaves extract have aromatic organic compounds (C=C) and aromatic amines (C-N) in addition to wormwood stems extract. We believe that the extra identified phytocompounds, at 80 °C, polymerized. During the polymerization process, the double bonds in the unsaturated hydrocarbon molecules are opened, which then combine to form a giant macromolecule. When the double bond is broken, a highly reactive atom (radical) is released, which has an unmatched electron. After that, the radical combines with another radical (both receiving paired electrons), thus starting the formation of a polymeric chain. It is well-known that the chain-growth polymerization involves the unsaturated monomers linking together, especially those containing carbon–carbon double bonds. Moreover, taking into account the preparation procedure of IONPs (detailed at Section 2.2), the method in which polymerization occurs is precipitation polymerization. This process begins initially in a continuous phase, as a homogeneous system where the reactants are completely soluble, but in the end, the formed polymer is insoluble and precipitates. The precipitation polymerization gives larger and irregulated particles, as a result of no stabilization [78,79]. In our case, when the aqueous suspensions based on WL 1_Fe_2_O_3_ NPs and WL 2_Fe_2_O_3_ NPs were formed, those were suspended for only a few minutes, then precipitated, and the supernatant above remained almost clear. This affirmation is sustained also by the increased PDI values, especially in the case of aqueous suspension based on IONPs obtained from wormwood leaves at 80 °C.

#### 3.1.7. XRF Analysis

To evaluate the metal traces present in the iron oxide NPs obtained starting from wormwood aqueous extract based on leaves (WL) and stems (WS), X-ray fluorescence analysis (XRF) was employed and the results are expressed in ppm, as presented in Table 3.

The XRF analysis reveals that none of the iron oxide NPs obtained contains traces of metals that could be noxious for medical use. The metals found in the samples, in insignificant quantities, may be due to the soil’s component, where the wormwood has grown and developed. It is worth noting that Fe has been found in all four samples, even in larger quantities in the samples obtained starting from wormwood aqueous extract based on stems.

The XRF analysis showed that the IONPs obtained by green synthesis could be used in biomedical applications because none of the samples obtained contains traces of metals which can be harmful in healthy living organism. This aspect is in agreement with other research studies, which affirm that nanoparticles based on iron oxides are biocompatible having a great attraction in biomedical treatments due to their non-toxicity [22,23,24,25,26,80,81,82].

### 3.2. In Vitro Screening of Fe_2_O_3_ NPs

Since the newly synthesized Fe_2_O_3_ NPs are addressed for biomedical purposes, establishing their biological profile is mandatory. Regarding this aspect, several in vitro techniques related to cell viability, cytotoxicity, and apoptosis have been implemented in the present study.

The in vitro assessment of the newly synthesized iron oxide NPs aimed to establish the biological profile of the samples by revealing the cytotoxic potential of the compounds on non-tumorigenic cells–immortalized human keratinocytes (HaCaT) and also to evaluate the antitumor activity induced by the samples on two different tumorigenic cell lines (A375 cells–amelanotic human melanoma and A431 cells–epidermoid human carcinoma cell line).

#### 3.2.1. Cell Viability Assessment by Means of Alamar Blue Colorimetric Test

**Figure 11:**
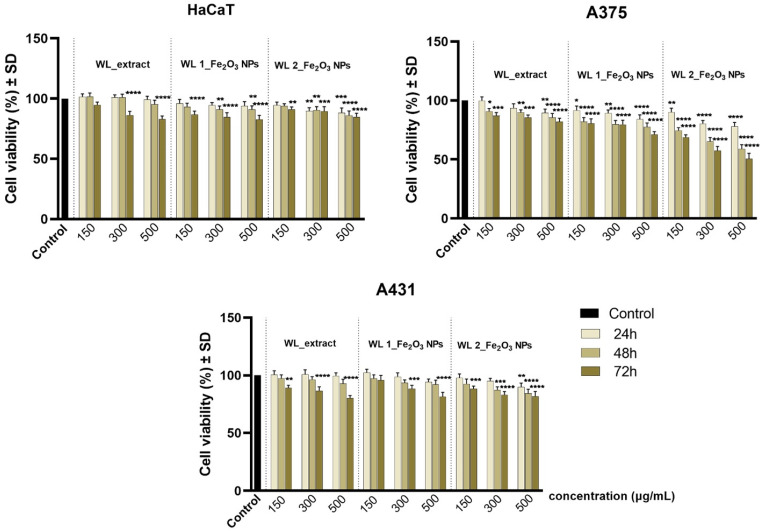
Cell viability percentage of immortalized human keratinocytes (HaCaT), human amelanotic melanoma cells (A375) and human epidermoid carcinoma cells (A431) after treatment with wormwood leaves extract (WL_extract) and two different Fe_2_O_3_ NPs obtained from this extract (WL 1_Fe_2_O_3_ NPs; WL 2_Fe_2_O_3_ NPs) at three different concentrations (150, 300, 500 µg/mL) for intervals of 24, 48, and 72 h. The viability percentage of sample-treated cells was normalized to the cell viability rate of control cells (cells without sample treatment). The data represent the mean values of three independent experiments ± standard deviation (SD). One-way ANOVA analysis was applied to determine the statistical differences followed by Dunnett’s multiple comparisons test (* *p* < 0.1; ** *p* < 0.01; *** *p* < 0.001; **** *p* < 0.0001 versus control cells).

**Figure 12:**
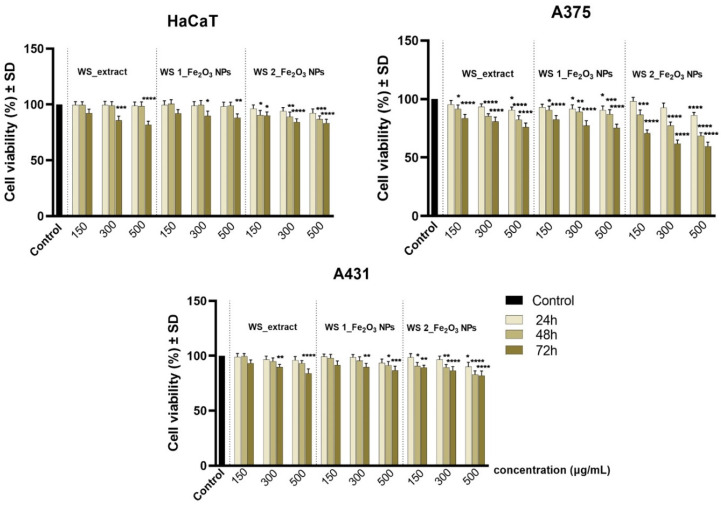
Cell viability percentage of immortalized human keratinocytes (HaCaT), human amelanotic melanoma cells (A375), and human epidermoid carcinoma cells (A431) after treatment with wormwood stems extract (WS_extract) and two different Fe_2_O_3_ NPs obtained from this extract (WS 1_Fe_2_O_3_ NPs; WS 2_Fe_2_O_3_ NPs) at three different concentrations (150, 300, 500 µg/mL) for intervals of 24, 48, and 72 h. The viability percentage of sample-treated cells was normalized to the cell viability rate of control cells (cells without sample treatment). The data represent the mean values of three independent experiments ± standard deviation (SD). One-way ANOVA analysis was applied to determine the statistical differences followed by Dunnett’s multiple comparisons test (* *p* < 0.1; ** *p* < 0.01; *** *p* < 0.001; **** *p* < 0.0001 versus control cells).

The preliminary in vitro profile was determined by one of the most consecrated colorimetric assays (Alamar Blue test). The method was performed to quantify the cell viability percentage of test cell cultures (HaCaT, A375, A431) after exposure to different concentrations (150, 300, 500 µg/mL) of samples for three time intervals—24 h, 48 h, and 72 h. The results obtained for wormwood leaves extract (WL_extract) and two different Fe_2_O_3_ NPs obtained from this extract (WL 1_Fe_2_O_3_ NPs; WL 2_Fe_2_O_3_ NPs) are presented in Figure 11, while the data recorded for wormwood stems extract (WS_extract) and two different Fe_2_O_3_ NPs resulting from it, WS 1_Fe_2_O_3_ NPs and WS 2_Fe_2_O_3_ NPs, are shown in Figure 12.

As a general observation of the data displayed in Figure 11, the cell viability rate of HaCaT, A375, and A431 decreased in a time-dependent manner, thus as the incubation time increased (from 24 h to 72 h), the percentage of viable cells decreased. However, a decreasing pattern of the viability rate was recorded as the concentration of the test compounds increased (from 150 to 500 µg/mL).

The cell viability rate of the healthy keratinocyte cell line (HaCaT) was not significantly affected when the cells were exposed to WL extract and two Fe_2_O_3_ NPs obtained from this extract (WL 1_Fe_2_O_3_ NPs; WL 2_Fe_2_O_3_ NPs), as the HaCaT viable population did not decrease beyond 80%.

Regarding the biological impact of test samples on the two tumorigenic cell lines (A375 and A431), the data revealed that human melanoma cell line–A375 cells elicited higher sensibility, compared to the human epidermoid carcinoma cell line–A431 cells. Thus, A375 cell population treated with 500 µg/mL of WL 1_Fe_2_O_3_ NPs reached a viable percentage of 84.21%, 77.58%, and 71.19% when implementing an exposure time of 24 h, 48 h, and 72 h, respectively. However, among all samples tested in the current study, WL 2_Fe_2_O_3_ NPs induced the most important cell viability decrease on A375 cells when applied at a concentration of 500 µg/mL, thus cell viability rates of 68.70%, 57.61%, 50.42% were recorded after 24 h, 48 h, and 72 h, respectively.

Whereas, compared to the effect observed on A375 cells, A431 cell culture treated with WL 1_Fe_2_O_3_ NPs (concentration of 500 µg/mL) manifested higher viability percentages, as follows: 94.37%, 92.24%, 81.59% at 24 h, 48 h, and 72 h post-treatment. The results obtained for A431 cells treated with 500 µg/mL of WL 2_Fe_2_O_3_ NPs were similar to the ones recorded for WL 1_Fe_2_O_3_ NPs, the cells reaching a viable rate of 81.90% after 72 h.

As presented in Figure 12, WS_extract induced a viability of 76.01% when applied to a concentration of 500 µg/mL, for an interval of 72 h. However, when WL_extract was applied under the same parameters (concentration of 500 µg/mL and exposure time of 72 h), A375 cells elicited a viable rate of 82.04% (Figure 11).

However, A375 cells manifested a viability of 82.57%, 77.28%, and 75.30% when treated for 72 h with WS 1_Fe_2_O_3_ NPs at concentrations of 150, 300, 500 µg/mL, respectively. Nevertheless, by applying the same concentrations (150, 300, 500 µg/mL) of WS 2_Fe_2_O_3_ NPs for an interval of 72 h, the cell viability percentages were more affected, A375 cells reaching viability rates of 70.72%, 61.87%, and 59.54%.

A431 cell viability was less affected after exposure to WS 1_Fe_2_O_3_ NPs and WS 2_Fe_2_O_3_ NPs, compared to A375 cell viability. For A431 cells, the most important cell viability decrease was obtained when the cells were exposed to WS 2_Fe_2_O_3_ NPs for 72 h, in this case the cells expressed viability percentages of 89.19%, 86.51%, and 81.95% when treated with concentrations of 150, 300, 500 µg/mL of WS 2_Fe_2_O_3_ NPs.

The preliminary in vitro assessment of Fe_2_O_3_ NPs by means of Alamar Blue method revealed that the cell viability of the healthy cell line (HaCaT) was not significantly affected after exposure to both wormwood extracts obtained from the leaves and stems of *Artemisia absinthium* L. or after treatment with Fe_2_O_3_ NPs resulting from them. As presented in Figure 11 and Figure 12, the viability percentage of HaCaT cell line did not decrease beyond 80%, even after exposure to the highest test concentration of 500 µg/mL for an interval of 72 h. Thus, according to the international standards regarding the biological assessment of medical devices (ISO Standard 10993-5: 2009) [83], a sample is considered cytotoxic if it induces a decrease of cell viability rate beyond 70%. Therefore, none of the test samples are considered cytotoxic for the healthy cell line (HaCaT).

Regarding the anti-tumor effect induced by the wormwood extracts, A375 cells treated with concentration of 500 µg/mL (for 72 h) were more affected after exposure to WS_extract than WL_extract, obtaining cell viability percentages of 76.01% versus 82.04%, respectively. However, the anti-tumor effect induced by both Fe_2_O_3_ NPs obtained from WS_extract on A375 cells proved a less pronounced anti-cancer activity compared to the Fe_2_O_3_ NPs obtained from WL_extracts (Figure 11 versus Figure 12).

#### 3.2.2. Cytotoxicity Evaluation via LDH Release Method

**Figure 13:**
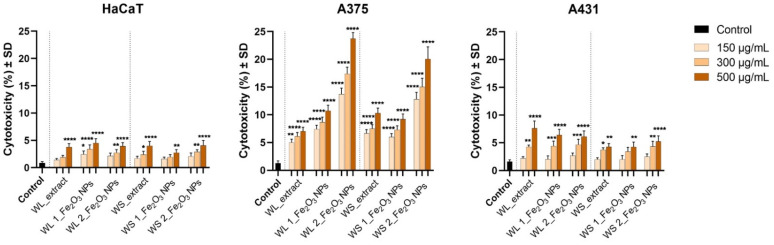
Cytotoxicity percentage of immortalized human keratinocytes (HaCaT), human amelanotic melanoma cells (A375), and human epidermoid carcinoma cells (A431) after treatment with wormwood leaves extract (WL_extract) and wormwood stems extract (WS_extract) and different Fe_2_O_3_ NPs obtained from these extracts (WL 1_Fe_2_O_3_ NPs; WL 2_Fe_2_O_3_ NPs, respectively WS 1_Fe_2_O_3_ NPs; WS 2_Fe_2_O_3_ NPs) at three different concentrations (150, 300, 500 µg/mL) for an interval of 72 h. The results represent the mean values of the cytotoxic rate of three separate experiments ± standard deviation (SD). One-way ANOVA analysis was applied to determine the statistical differences followed by Dunnett’s multiple comparisons test (* *p* < 0.1; ** *p* < 0.01; *** *p* < 0.001; **** *p* < 0.0001 versus control cells).

In order to complete the preliminary screening tests on HaCaT, A375 and A431 cell lines, the LDH release assay was employed when treating the cells with concentrations of 150, 300, 500 µg/mL, at 72 h post-exposure, where the highest cell viability decrease was observed via Alamar Blue test. The results obtained are presented in Figure 13.

The cytotoxicity rate recorded for HaCaT cells treated with test samples did not exceed the rate of 4.5% when exposed to the high concentration of 500 µg/mL. However, A375 elicited a cytotoxicity rate of 10.71% and 23.73% after exposure to 500 µg/mL of WL 1_Fe_2_O_3_ NPs and WL 2_Fe_2_O_3_ NPs, whereas A375 cell cultures manifested less cytotoxicity when treated with WS 1_Fe_2_O_3_ NPs and WS 2_Fe_2_O_3_ NPs, as follows: 9.21% and 20.06%.

Regarding the cytotoxicity rate expressed by A431 cells, among all test samples, WL_extract induced the higher cytotoxic effect of 7.62%, WL 1_Fe_2_O_3_ NPs and WL 2_Fe_2_O_3_ NPs showing lower cytotoxicity rates of 6.43% and 6.13%, respectively. WS_extract and both Fe_2_O_3_ NPs obtained from it (WS 1_Fe_2_O_3_ NPs and WS 2_Fe_2_O_3_ NPs) induced even lower cytotoxicity, around 5%.

Nevertheless, the results obtained through LDH release method corroborates with the data revealed by the Alamar Blue test (Figure 11 and Figure 12), respecting the same cytotoxic pattern of the test samples. Moreover, the amount of LDH leaked extracellular was higher when A375 cells were exposed to WS_extract, compared to WL_extract (10.28% versus 7.03%).

#### 3.2.3. Apoptotic Markers Detection through 4′,6-Diamidino-2-Phenylindole (DAPI) Staining

**Figure 14:**
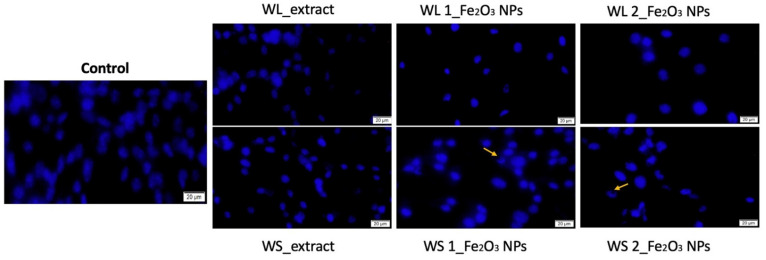
Morphological aspects of immortalized human keratinocytes-HaCaT nuclei treated with concentration of 500 µg/mL of both wormwood extracts: leaves (WL_extract) and stems (WS_extract) and different Fe_2_O_3_ NPs obtained from these extracts (WL 1_Fe_2_O_3_ NPs; WL 2_Fe_2_O_3_ NPs, respectively WS 1_Fe_2_O_3_ NPs; WS 2_Fe_2_O_3_ NPs) for an interval of 72 h. The scale bars represent 20 μm. Fe_2_O_3_ NPs that are either attached or embedded in the cells nuclei are marked with yellow arrows.

**Figure 15:**
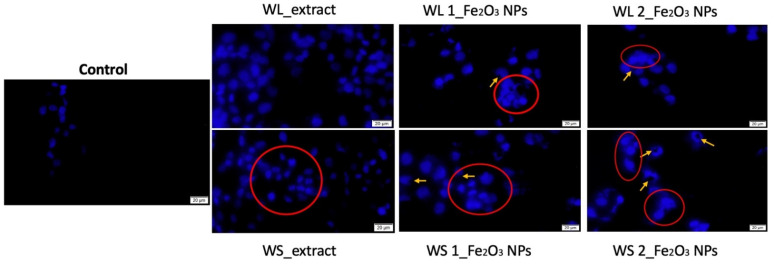
Morphological aspects of human melanoma cells–A375 nuclei treated with concentration of 500 µg/mL wormwood leaves (WL) and wormwood stems (WS) extracts and different Fe_2_O_3_ NPs obtained from these extracts (WL 1_Fe_2_O_3_ NPs; WL 2_Fe_2_O_3_ NPs, respectively WS 1_Fe_2_O_3_ NPs; WS 2_Fe_2_O_3_ NPs) for an interval of 72h. The scale bars represent 20 μm. Fe_2_O_3_ NPs that are either attached or embedded in the cells nuclei are marked with yellow arrows, while the red circles highlight the apoptotic markers.

To provide an insight into the cytotoxic effect induced by test samples on cell nucleus integrity, the parameters (concentration of 500 µg/mL and 72 h time interval) that triggered the highest levels of LDH, were also implemented for DAPI staining, in order to quantify the apoptosis potential of the samples, by using A375 cell line where the most important cytotoxic effect was detected. Even though healthy human keratinocytes (HaCaT) manifested no intense cytotoxic effect when LDH method was employed, HaCaT cells were used as control for apoptosis quantification between the healthy and tumorigenic cell lines (HaCaT versus A375). The results obtained for HaCaT cells are presented in Figure 14, while the results obtained for A375 are presented in Figure 15.

As presented in Figure 14, HaCaT cells treated with test samples at concentration of 500 µg/mL for 72 h, did not manifest important signs of apoptosis, such us: chromatin condensation, nuclear membrane blebbing, or DNA fragmentation. As captured on images, HaCaT nuclei presented normal morphological organization, with large nucleus and visible nucleolus. Moreover, cells nuclei showed uniform chromatin distribution.

A375 cells treated with concentration of 500 µg/mL of test samples expressed important apoptosis features at 72 h post-treatment, as depicted in Figure 15. The most significant apoptosis markers are represented by chromatin condensation and are highlighted with red circles. Moreover, the images revealed that several Fe_2_O_3_ nanoparticles were either attached or embedded within A375 cells nuclei, as indicated by the yellow arrows from Figure 15. This aspect may be explained by the different polyphenolic composition of the *Artemisia absinthium* L. leaves and steams extracts, WS_extract containing a higher amount of chlorogenic acid, isoquercitrin and rutin, compared to WL_extract, as detected by LC-MS analysis in a previous article published by our group [77].

In summary, the data recorded via LDH release method and DAPI staining (Figure 12 and Figure 13) corroborate the results obtained through Alamar Blue test and highlight the non-toxicological potential of the samples for the non-tumorigenic immortalized human keratinocytes (HaCaT), when applied to concentrations up to 500 µg/mL for 72 h.

Even though the biological features of the phytomediated Fe_2_O_3_ NPs are closely related to the functional moieties, such as alkaloids and flavonoids that act as reducing agents in the synthesis process of the Fe_2_O_3_ NPs [23], another important aspect that plays a key role in the cytotoxic effect induced by Fe_2_O_3_ NPs is related to the accumulation rate of Fe_2_O_3_ NPs within the cell nuclei, as already reported by Narayanan and Han [22]. Thereby, the higher cytotoxic effect induced by WS 1_Fe_2_O_3_ NPs and WS 2_Fe_2_O_3_ NPs may be explained by the higher accumulation rate within the A375 cells nuclei, compared with the accumulation rate of WL 1_Fe_2_O_3_ NPs and WL 2_Fe_2_O_3_ NPs (as noticed through DAPI staining in Figure 15). Moreover, the uptake ratio of Fe_2_O_3_ NPs may be closely related to the hydrodynamic diameter of the NPs, as WS 1_Fe_2_O_3_ NPs and WS 2_Fe_2_O_3_ NPs presented a nanosized diameter of 244 and 309 nm, respectively, whereas the samples with a low accumulation rate within the cells (WL 1_Fe_2_O_3_ NPs and WL 2_Fe_2_O_3_ NPs) have a diameter within micrometric scale of 8.943 and 1.048 μm (Table 2).

There are several routes that may be involved into the intracellular up-taking process of the iron oxide NPs, such as macro-pinocytosis and endocytosis-mediated pathways [84]. However, the accumulation rate is mainly influenced by the physicochemical features of the NPs, such as surface charge, shape, and size [85].

Still, the up-taking pathway involved in the intracellular accumulation process of the newly synthetized Fe_2_O_3_ NPs is an important aspect that may provide relevant data to the in vitro cytotoxic profile of the plant-mediated Fe_2_O_3_ NPs and should be further investigated within upcoming studies.

## 4. Conclusions

The present study reports a facile green synthesis method to obtain ecofriendly iron oxide NPs, by employing two aqueous wormwood extracts (obtained from leaves and stems of *Artemisia absinthium* L.) used as reducing agents. The morphology and particle size are two important parameters that must be taken into account when preparing IONPs based on a plant extract. Besides these parameters, it is often needed to adjust the reaction conditions (reaction time, metallic precursor salts concentration, pH, temperature, or controlled atmosphere). Even so, it cannot be predicted the type of nanoparticles obtained, until a proper investigation and identification technique is performed. According to electron microscopy investigation, the as synthetized iron oxide NPs are extremely small (4.7 ± 0.8 nm from SEM and 2.8 ± 0.9 nm from TEM), agglomerated and quasi-spherical in shape. The hydrodynamic diameter revealed that the aqueous suspensions with IONPs obtained from leaves are of the microns order (denoting giant clusters) as against aqueous suspensions with IONPs obtained from stems are in the nanometric scale.

The newly synthetized iron oxide NPs showed promising potential for biomedical applications, as the preliminary in vitro screening revealed that the phyto-mediated Fe_2_O_3_ NPs did not show cytotoxic activity on non-tumorigenic immortalized human keratinocytes (HaCaT), while the tumorigenic cell lines, especially A375 cells are significantly affected, the cells releasing a high amount of extracellular LDH and specific signs of apoptosis when exposed to concentration of 500 µg/mL for an interval of 72 h.

## Figures and Tables

**Table 1:** The size distribution particles as a function of counts.

Sample	Diameter [nm]	Total Counts
WL 1_Fe_2_O_3_ NPs	3.1 ± 1.0	59
WL 2_Fe_2_O_3_ NPs	2.9 ± 0.8	31
WS 1_Fe_2_O_3_ NPs	3.0 ± 1.0	51
WS 2_Fe_2_O_3_ NPs	2.3 ± 0.5	83

**Table 2:** Characteristics of the aqueous suspensions.

Aqueous Suspension	Hd [nm]	PDI
WL 1_Fe_2_O_3_ NPs	8943	0.689
WL 2_Fe_2_O_3_ NPs	1048	0.473
WS 1_Fe_2_O_3_ NPs	244	0.449
WS 2_Fe_2_O_3_ NPs	309	0.366

**Table 3:** Metals composition (ppm) of iron oxide NPs.

Sample	Ca	Ti	Cr	Mn	Fe	Ni	Cu	Zn
WL 1_Fe_2_O_3_ NPs	0.074	0.015	0.022	0.109	**45.819**	0.005	0.007	0.004
WL 2_Fe_2_O_3_ NPs	0.004	0.015	0.021	0.118	**45.871**	0.006	0.005	0.004
WS 1_Fe_2_O_3_ NPs	-	0.009	0.032	0.051	**54.591**	0.007	0.006	0.005
WS 2_Fe_2_O_3_ NPs	0.061	0.010	0.026	0.144	**58.872**	0.006	0.003	0.003

## Data Availability

Not applicable.
